# Is Single-stage Revision According to a Strict Protocol Effective in Treatment of Chronic Knee Arthroplasty Infections?

**DOI:** 10.1007/s11999-014-3721-8

**Published:** 2014-06-13

**Authors:** Fares Sami Haddad, Mohamed Sukeik, Sulaiman Alazzawi

**Affiliations:** Department of Trauma and Orthopaedics, University College London Hospital, 235 Euston Road, London, NW1 2BU UK

## Abstract

**Background:**

The increasing number of patients experiencing periprosthetic total knee arthroplasty (TKA) infections and the cost of treating them suggest that we seek alternatives to two-stage revision. Single-stage revision is a potential alternative to the standard two-stage procedure because it involves only one surgical procedure, so if it is comparably effective, it would be associated with less patient morbidity and lower cost.

**Questions/purposes:**

We compared (1) the degree to which our protocol of a highly selective single-stage revision approach achieved infection control compared with a two-stage revision approach to TKA infections; and (2) Knee Society scores and radiographic evidence of implant fixation between the single-stage and two-stage patients who were treated for more complicated infections.

**Methods:**

Between 2004 and 2009, we treated 102 patients for chronic TKA infections, of whom 28 (27%) were treated using a single-stage approach and 74 (73%) were treated using a two-stage approach. All patients were available for followup at a minimum of 3 years (mean, 6.5 years; range, 3–9 years). The indications for using a single-stage approach were minimal/moderate bone loss, the absence of immunocompromise, healthy soft tissues, and a known organism with known sensitivities for which appropriate antibiotics are available. Participants included 38 men and 64 women with a mean age of 65 years (range, 45–87 years). We used the Musculoskeletal Infection Society definition of periprosthetic joint infection to confirm infection control at the last followup appointment. Radiographs were evaluated for signs of loosening, and patients completed Knee Society Scores for clinical evaluation.

**Results:**

None of the patients in the single-stage revision group developed recurrence of infection, and five patients (93%) in the two-stage revision group developed reinfection (p = 0.16). Patients treated with a single-stage approach had higher Knee Society scores than did patients treated with the two-stage approach (88 versus 76, p < 0.001). However, radiographic findings showed a well-fixed prosthesis in all patients with no evidence of loosening at last followup in either group.

**Conclusions:**

Our data provide preliminary support to the use of a single-stage approach in highly selected patients with chronically infected TKAs as an alternative to a two-stage procedure. However, larger, multicenter, prospective trials are called for to validate our findings.

**Level of Evidence:**

Level III, therapeutic study. See Instructions for Authors for a complete description of levels of evidence.

## Introduction

Most TKA studies today report infection in fewer than 2% of primary and 5% of revision procedures [16, 22, 34, 35]. Nevertheless, both diagnosis and management of periprosthetic TKA infections remain challenging because the ability to detect and eradicate pathogens in periarticular structures and the magnitude of the host response to infection vary with the virulence of the infecting organism and the immunocompetence of the host [12]. Management depends on a number of factors including the acuteness or chronicity of the infection, the infecting organism and its sensitivity profile to antibiotics, the health of the patient, the fixation of the prosthesis, available bone stock, and the particular philosophy and training of the surgeon [12, 23, 37].

Two-stage revision remains the standard for treatment of chronic TKA infections because many series report the successful eradication of a periprosthetic joint infection (PJI) in more than 90% of patients using this approach [12, 16, 38]. Furthermore, it permits the use of allografts, which is particularly important given the frequency of femoral and tibial defects associated with TKA infections [14, 17]. Nevertheless, this procedure is costly, time-consuming, and may result in increased damage to bone and surrounding soft tissues [35].

Single-stage revision in selected cases has become an appealing alternative because it involves only one surgical procedure and, if comparably effective, will be associated with less patient morbidity and potentially improved functional outcomes and less expense [11, 24, 35]. Infection control using a single-stage strategy in selected patients is achieved in 67% to 95% of patients [4, 10, 20, 30–32, 36].

At our institution, we carry out single-stage TKA revisions for chronic infections in very selected circumstances and, therefore, we determined in this study (1) the degree to which our protocol of a highly selective single-stage revision approach achieved infection control compared with a two-stage revision approach to TKA infections; and (2) Knee Society scores and radiographic evidence of implant fixation between the single-stage and two-stage patients who were treated for more complicated infections.

## Patients and Methods

Between 2004 and 2009, we treated 102 patients for chronic TKA infections, of whom 28 (27%) were treated using a single-stage approach and 74 (73%) were treated using a two-stage approach. All patients were available for followup at a minimum of 3 years (mean, 6.5 years; range, 3–9 years).

In the two-stage revision group, 12 patients had undergone two and 24 undergone one previous aseptic revision. There were no prior revision procedures in the remaining 38 patients. In the single-stage group, eight patients had undergone aseptic revisions and the rest were primaries.

At our institution, a patient with suspected TKA infection is promptly referred to the knee surgeons who deal with PJIs regularly because this is a specialized procedure and there is no role for simple incision and drainage or repetitive washouts, which result in emergence of resistant microorganisms [35]. Clinical presentation (pain, fever, swelling, skin redness, discharging sinus), serologic testing (erythrocyte sedimentation rate [ESR] > 30 mm/hour; C-reactive protein [CRP] > 10 mg/L), knee aspiration, and biopsy samples help us diagnose PJI [33, 35]. Definitive diagnosis, however, is established when three to six specimens are sampled from different sites at the time of surgery (eg, capsule, femur and tibia) and the same microorganism is cultured from at least three specimens [2, 35, 37].

A decision to perform surgery was based on either growing a microorganism from the tissue aspiration/biopsies or presence of a sinus tract communicating with the prosthesis. A microorganism was identified preoperatively in all single-stage patients and in 65 of the two-stage patients, whereas the remaining nine patients were identified postoperatively only despite the presence of a discharging sinus in five patients. The remaining four patients had compelling evidence of PJI with elevated inflammatory markers, loose prostheses, and purulence on aspiration of the joints despite the absence of an isolated microorganism.

We graded all patients according to a standardized protocol for chronic hip and knee PJIs based on the criteria previously set out by Haddad et al. [13] and considered them for either a single- or two-stage revision procedure accordingly (Table 1). The indications for using a single-stage approach during the period in question included (1) insignificant bone loss (eg, Anderson Type I and II defects [6, 7]) or a soft tissue defect that could be closed primarily; (2) nonimmunosuppressed hosts: patients who are not rheumatoid or diabetic or on immunosuppressant medication and did not have ongoing sepsis elsewhere or chronic disease such as anemia or cancer; and (3) isolation of a single low virulent organism preoperatively, which is sensitive to bactericidal antibiotic treatment. Hence, we excluded polymicrobial infections and multiresistant organisms such as methicillin-resistant *Staphylococcus aureus* and methicillin-resistant *Staphylococcus epidermidis* and included appropriate patients only after discussion with our microbiologist colleagues. If patients had any of the contraindications (Table 1), they underwent a two-stage revision instead.

**Table 1:** Contraindications for single-stage revision THA and TKA

Category	Compromising factor
Local	Significant soft tissue compromise Significant bone loss precluding cemented reconstruction Peripheral vascular disease
Host	Immunosuppression Concurrent sepsis Systemic disease Reinfection
Organism	Multiresistant organisms MRSA/MRSE Polymicrobial infection Unusual commensals Unusual resistance profiles Unidentified infective organisms

Reproduced with permission and copyright © of the British Editorial Society of *Bone and Joint Surgery* [Oussedik SI, Dodd MB, Haddad FS. Outcomes of revision total hip replacement for infection after grading according to a standard protocol. *J Bone Joint Surg Br.* 2010;92:1222–1226]; MRSA = methicillin-resistant *Staphylococcus aureus*; MRSE = methicillin-resistant *Staphylococcus epidermidis.*

Participants included 28 patients in the single-stage group with a mean age of 63 years (range, 48–87 years) and equal distribution of 14 women and men. On the other hand, the two-stage group included 74 patients with a mean age of 68 years (range, 45–85 years) of whom 41 were women and 33 were men. Overall there were 12 patients with sinus tracts communicating with the prosthesis all in the two-stage group. No bilateral infections were included in our study. No patient had a history of infection of the affected knee. The majority of patients had osteoarthritis as the underlying pathology for their primary TKA (74 patients) followed by inflammatory arthropathy (20 patients) and posttraumatic/acute vascular necrosis resulting in secondary osteoarthritis in eight patients. In patients who had undergone revision TKA, the original indications for reoperation after their primary procedures were aseptic loosening and wear. Comorbidities were assessed according to the American Society of Anesthesiologists grading system [19]; nine patients were Grade I, 56 Grade II, and 37 Grade III. Three patients died during the followup period but had a minimum of 2 years’ data available for analysis. No patients were recalled specifically for this study; all data were obtained from medical records and radiographs.

### Surgical Technique: Single-stage Revision

The operation consists of open aggressive débridement with removal of all components and cement, during which multiple samples are sent to microbiology before administration of antibiotics and the knee is irrigated with hydrogen peroxide and Betadine^®^ solutions (Videne, Ecolab Ltd, Swindon, UK) and pulsatile lavage. The wound is then soaked in aqueous Betadine^®^ and the wound edges are approximated. The patient is then redraped, the surgical team rescrubs, and new instruments are used. After a further lavage, implantation of a new prosthesis is performed using antibiotic-loaded cement (ALC) according to known sensitivities at a volume of < 5% of the total weight of cement powder. For example, we commonly used 1 g vancomycin and 1 g gentamicin per 40-g bag of Palacos^®^R (Heraeus Medical, Wehrheim, Germany) for our single-stage revisions. Postoperatively, patients continue antibiotic therapy tailored to the sensitivities of intraoperative cultures for at least 6 weeks until inflammatory markers (CRP, ESR) and nutritional markers such as plasma albumin concentration return to stable limits (levels normalized in 90% of cases). Normal levels were defined as an ESR < 30 mm/hour, CRP < 10 mg/L, and albumin 35 to 50 g/L. The change from intravenous to oral therapy is effected as soon as we have a full organism sensitivity profile and after consultation with our infectious diseases team with whom we have a fortnightly multidisciplinary meeting (IV antibiotics for 1 week: four patients, 2 weeks: seven patients, 6 weeks: 17 patients). Long-term oral suppressive antibiotic therapy was not used in any patients after IV treatment had concluded.

### Surgical Technique: Two-stage Revision

Intraoperatively, the first part of the operation is similar to a single-stage revision. However, after rescrubbing and redraping, a temporary articulating ALC spacer is implanted instead. This spacer normally contains 3 g vancomycin and 2 g gentamicin per sachet of Palacos^®^R (Heraeus Medical), which provides a broad spectrum of coverage for organisms commonly encountered with deep periprosthetic infections while reducing the development of resistant strains [1]. Postoperatively, the patient is allowed to mobilize partial weightbearing with crutches and is discharged home when deemed safe. All patients had IV antibiotics for the first 5 days and then either IV or oral antibiotic therapy was continued and tailored to the sensitivities of intraoperative cultures and continued for 6 weeks (seven patients had 2 weeks of IV and then oral antibiotics, five had 6 weeks of IV antibiotics). The decision to proceed with insertion of a new prosthesis is determined by the clinical response of the patient including wound healing and inflammatory and nutritional markers indicating resolution of infection, which is confirmed after 2 weeks of discontinuing any antibiotics the patient was taking. At the second stage, the spacer is removed and the underlying cement mantle is fragmented and removed piecemeal without sacrificing bone stock. An appropriate prosthesis is then reimplanted with cemented components, and allografts may be used in cases of severe bone loss. Types of implants and augments used are listed (Table 2).

**Table 2:** Types of implants/reconstructions used for the single- and two-stage revisions of infected TKAs

Type of implant/reconstruction	Number of single-stage revisions	Number of two-stage revisions
Augments	4	9
Cones	2	5
Stems on one side or both	28	74
Semiconstrained implants	18	50
Hinges	7	19
Bone graft	0	6

Regardless of the treatment strategy followed, we review all our patients postoperatively at 6 weeks, 6 months, 1 year, and then on a yearly basis looking for clinical symptoms and signs of infection as well as CRP and ESR. One of us (FSH) performed all the procedures. We obtain plain radiographs including AP, lateral, and skyline views of both knees at every followup appointment. We assess component position, radiolucencies/osteolysis, and loosening according to The Knee Society recommendations [8, 29]. Distinguishing infective loosening from aseptic loosening radiographically can be difficult; however, signs of an infected knee arthroplasty include progressively enlarging lucencies, endosteal scalloping, periostitis, and focal lysis [29].

Control of infection is defined as absence of clinical, serologic, and radiographic signs of infection and absence of death secondary to infection or treatment during the followup period. We used the Musculoskeletal Infection Society criteria in our last outpatient review to assess and confirm infection control [26, 27]. We define failure as any major operation performed in any subgroup of patients for control of infection, including a two-stage revision, excision arthroplasty, arthrodesis, and amputation, or the need for long-term antibiotic suppression. We consider reinfection to be an infection with the same or another organism. The mean interval time between each stage was 62 days (range, 42–119 days). Duration of antibiotic treatment was 63 days (range, 42–85 days) for the single-stage group and 12 days (range, 5–42 days) for the two-stage group.

**Fig. 1:**
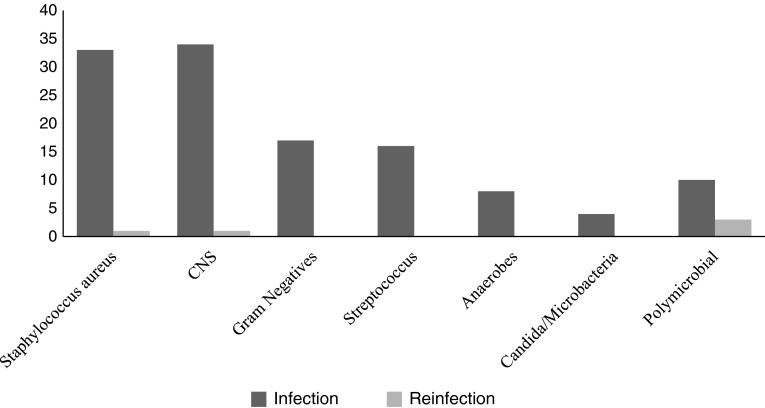
Microorganisms responsible for infections and reinfections are shown. CNS = coagulase-negative *Staphylococcus.*

The causative microorganism was identified preoperatively in all single-stage patients and in 65 of the two-stage patients, whereas the remaining nine patients were identified postoperatively. Microbiology from intraoperative tissue sampling confirmed bacterial infection in all patients with the most commonly isolated organism being coagulase-negative *Staphylococcus* (34 patients [33%]) of which nine were methicillin-resistant followed by *S aureus* (33 patients [32%]), of which 11 were methicillin-resistant (Table 3). Other microorganisms isolated included Gram-negatives (17 patients), *Streptococcus* (16 patients), anaerobes (eight patients), and *Candida* and *Mycobacteria* (four patients). Ten patients had polymicrobial infections. Most common reinfections were the result of polymicrobial infections (Fig. 1).

**Table 3:** Microorganisms grown from intraoperative tissue biopsies

Microorganism	Number of single-stage revisions	Number of two-stage revisions
*Staphylococcus aureus* (methicillin-resistant *S. aureus*)	8 (0)	25 (11)
Coagulase-negative *Staphylococcus*	11	23
(methicillin-resistant *Staphylococcus epidermidis*)	(0)	(9)
*Streptococcus*	4	12
Gram-negatives	4	13
Anaerobes	1	7
*Candida/Mycobacteria*	0	4
Polymicrobial	0	10

The functional outcome for all patients was evaluated using the Knee Society scoring system, which was recorded preoperatively and at the 2-year followup.

Statistical analysis was carried out using the two-sample t-test or Mann-Whitney U test for continuous outcomes and a chi-square test or Fisher’s exact test for categorical outcomes.

## Results

None of the patients in the single-stage revision group developed recurrence of infection, and five patients (7%) in the two-stage revision group developed reinfection (p = 0.16). Those patients, however, underwent a further two-stage revision procedure and had their infections controlled at last followup.

The Knee Society score was higher in the single-stage group at 2 years than in the two-stage group (mean, 88; range, 38–97 versus 76; range, 29–93; p < 0.001). Both groups improved in this score after successful reconstruction from a mean of 32 (range, 18–65) to a mean of 88 (range, 38–97) in the single-stage group and 31 (range, 17–70) to 76 (range, 29–93) in the two-stage group (Table 4). Radiographic findings showed a well-fixed prosthesis in all patients of both groups with no evidence of loosening at the most recent followup.

**Table 4:** Knee Society scores and visual analog scale satisfaction scores

Outcomes	Single-stage	Two-stage	p value
Number of patients	28	74	N/A
Recurrent infection	0	5	< 0.01
KSS preoperatively	32 (18–65)	31 (17–70)	NS
KSS at 2 years	88 (38–97)	76 (29–93)	< 0.02
Difference in KSS	56	45	< 0.02
Visual analog scale at 2 years	7.82	6.18	< 0.01

Ranges in parentheses; KSS = Knee Society score; N/A = not applicable; NS = not significant.

## Discussion

Despite the relatively low rates of PJIs after TKAs, they remain a leading cause of revision surgery as a result of an ever increasing number of knee arthroplasties performed yearly for an aging population [12, 35]. In contrast to two-stage revisions, single-stage surgery may offer a shorter hospital stay, the avoidance of complications associated with a second operation, improved postoperative function and pain, and lower cost; however, whether infection control is sacrificed for these endpoints remains controversial, and if it is, a single-stage approach would likely not be justified. In this study, we therefore determined (1) the degree to which our protocol of a highly selective single-stage revision approach achieved infection control compared with a two-stage revision approach to TKA infections; and (2) Knee Society scores and radiographic evidence of implant fixation between the single-stage and two-stage patients who were treated for more complicated infections.

Our study is associated with some limitations. First, a single-stage revision procedure was applied in a highly selected patient population using the indications we have defined (Table 1) and is not suitable for “all comers.” Second, patients undergoing two-stage procedures tend to have been more complicated taking into consideration that they had undergone multiple revision procedures and had less bone stock to start off with, which may account for the more complex reconstructions and the higher observed Knee Society scores in the single-stage patients. Third, 3 years of followup is not sufficient to know that these patients will remain without infections; there is a risk of infection recurring, and hence our close followup continues for this cohort of patients. Fourth, infection control after knee arthroplasties can be affected by a number of risk factors, including age, sex, time from operation, duration of symptoms, patient comorbidities, and the pathogen causing the infection [5, 12, 35]. Because of the small number of patients within each subgroup, the heterogeneity of the study population (type of original operation, number of previous surgeries, and type of surgery performed) and the observational nature of this study, we were unable to perform a multivariate analysis to further investigate the effect of those risk factors on infection control outcome. Fifth, despite no recurrence of infection in the single-stage group of patients, the numbers included in this study remain small. This, however, reflects the difficulty of finding large numbers suitable for a single-stage revision even at a tertiary center dealing with significant numbers of periprosthetic infections.

Our results for infection control using two-stage revision for chronic infections are consistent with those previously reported in the literature, especially where a clear protocol has been followed [3, 9, 12, 15, 16, 18, 21, 28, 38]. It is of note, however, that the inclusion and exclusion criteria as well as management protocols varied among those studies, occasionally including all four types of periprosthetic infections rather than chronic infections only. Additionally, some of the studies did not differentiate between knees and hips when reporting their results, which resulted in a wide range of infection control rates. On the other hand, single-stage revisions for chronic infections are regaining momentum and our results certainly reflect a strict protocol, which has led to superior results to what has been reported in the literature [4, 10, 20, 30–32, 36] (Table 5). The only study with equivalent results to our study reporting 100% infection control with a single-stage strategy was recently published by Parkinson et al. [25]. However, in their 12-patient series, they did not mention details about the inclusion criteria for their protocol apart from growing a microorganism from the arthroscopy performed preoperatively for a diagnosis of infection. Additionally, there are no details regarding the type of infection treated (acute or chronic, postoperative or hematogenous).

**Table 5:** Previous studies reporting infection control after single-stage revision for infected TKAs

Study	Number of cases	Infection control (%)	Followup (years)
Buechel et al., 2004 [4]	22	90.9	10.2
Goksan and Freeman, 1992 [10]	18	88.8	5
Lu et al., 1997 [20]	8	87.5	1.7
Silva et al., 2002 [30]	37	89.2	4
Singer et al., 2012 [31]	63	95	3
Sofer et al., 2005 [32]	15	93	1.5
von Foerster et al., 1991 [36]	104	73.1	6.3

Other studies also reported improvement in Knee Society scores after a single-stage revision for PJI. For example, Singer et al. [31] reported a mean Knee Society score of 72 points after 24 months and a mean reported range of movement of 104°. Buechel et al. [4] also had a similar mean final postoperative knee score of 79.5 (range, 35–94). This may support an easier convalescence as a potential advantage of a single-stage procedure, especially with no differences found in prosthesis fixation as seen in our current study at the latest followup.

In conclusion, our data support the use of single-stage revision surgery in chronic TKA infections as an alternative to a two-staged procedure with high infection control rates when patients are carefully selected. However, larger, multicenter, prospective trials are called for to validate our findings.
